# The complete chloroplast genome of a distinctive fern, *Coniogramme intermedia* Hieron. (Pteridaceae)

**DOI:** 10.1080/23802359.2021.1994892

**Published:** 2021-11-03

**Authors:** Danke Zhang, Wenli Yang, Hao Liu, Yueming Cui, Lei Wang, Xuehua Liu, Gangmin Zhang

**Affiliations:** a Laboratory of Systematic Evolution and Biogeography of Woody Plants, School of Ecology and Nature Conservation, Beijing Forestry University, Beijing, China; b College of Landscape Architecture and Tourism, Hebei Agricultural University, Baoding, China; c College of Horticulture and Plant Protection, Henan University of Science and Technology, Luoyang, China

**Keywords:** Complete chloroplast genome, phylogeny, Pteridaceae, *Coniogramme*

## Abstract

*Coniogramme intermedia* Hieron. is a morphologically distinctive species in the genus. It is identified by lanceolate pinnules with serrated margins, free veins, hydathodes extending into teeth, and laminae abaxially hairy. It is mainly distributed in the tropical and subtropical regions of Asia. Herein, we report the first complete chloroplast genome sequence of *C. intermedia.* Also, it is the opening one of the genus *Coniogramme* Fée. The chloroplast genome sequence is 153,561 bp in length. The genome has a typical quadripartite structure, including a large single-copy (LSC) region of 82,817 bp, a small single-copy (SSC) region of 21,236 bp, and two inverted repeat (IR) regions of 24,754bp each. The total GC content is 45.0%. The complete plastome sequence contains 114 genes, including, 81 protein-coding, 29 tRNA, and four rRNA genes. The phylogenetic analysis of Pteridaceae based on the complete chloroplast genomes was also presented in this study.

*Coniogramme* Fée is a fern genus with about 50 species worldwide, which belongs to the subfamily Cryptogrammoideae in Pteridaceae (Christenhusz et al. 2011; Zhang and Ranker 2013; PPG I 2016). It is characterized by its large habit with creeping rhizomes and 1–3 pinnate fronds with exindusiatesori borne along the lateral veins. However, the genus is poorly differentiated and has long been one of the most problematic fern groups with respect to its specific definition. Fraser-Jenkins (2008) concluded that the taxonomy of *Coniogramme* is very complicated and has been confusing taxonomists in species circumscription.

This genus was divided into two sections, and sect. *Coniogramme* further divided into ser. *Serratae* and ser. *Fraxineae* (Lu 2001). *Coniogramme* ser. *Serratae* is characterized by pinnules with serrated margins, distinguishing from entire margins in *C.* ser. *Fraxineae*. Among ser. *Serratae*, *Coniogramme intermedia* Hieron. is the most wildly distributed one in East Asia, including China, India, Japan, Korea and Vietnam. It is distinctive by lanceolate pinnules with serrated margins, free veins, hydathodes extending into teeth, and laminae abaxially hairy. In order to better understand this species, we report and annotate the first complete chloroplast genome of *C. intermedia* using the next-generation sequencing method.

Fresh young leaves of *C. intermedia* were collected from Longchuan County, Yunnan province, China (N97°59′47.10″, E24°13’5.38”). Voucher specimens were deposited in the Herbarium of Beijing Forestry University (BJFC) (under collection number of *PT_954*). CTAB method (Doyle and Doyle 1987) was used to extract the total genome DNA which was then send to Meiji Company (Meiji Biotech Co. Ltd., Beijing) for 2 × 150 bp pair end sequencing using Illumina HiSeq 4000 platform. Referring to the published plastid genome of *Cryptogramma acrostichoides* R. Br. (NC_040211) and *Llavea cordifolia* Lag. (NC_040216), the Map to Reference function of Geneious R11 (Kearse et al. 2012) was used to filter available reads. In total, 14 Gb of 150-bp clean reads were generated. These available reads were *de novo* assembled and concatenated into larger ones using Geneious R11. The original data were again mapped to the larger contigs to extend their boundaries until all contigs were able to concatenate to one contig. The IR region was determined using the Repeat Finder function in Geneious R11. The assembled chloroplast sequence was annotated using the Plastid Genome Annotator (PGA, Qu et al. 2019), and verified by Geneious R11.

The complete chloroplast genome sequence of *C. intermedia* is 153,561 bp in length, with a large single-copy (LSC) region of 82,817 bp, a small single-copy (SSC) region of 21,236 bp, and two inverted repeats (IR) of 24,754 bp. The plastome contains 114 functional genes, including 81 protein-coding genes, 29 tRNA genes, and four rRNA genes. The total sequence GC content is 45.0%. Structural variations such as gene inversions, transpositions, and IR expansion of this plastome sequence was similar to that of other samples in Cryptogrammoideae (Robison et al. 2018).

*C. intermedia* and other 13 species from Pteridaceae, with two species from eupolypods as outgroup species, were used for phylogenetic analysis (Figure 1). A total of 16 complete chloroplast genomes were aligned using MAFFT (Katoh et al. 2017). The maximum likelihood (ML) method was used for phylogeny reconstruction. The sequence alignment was followed Liu et al. (2018). Then, the ML tree was constructed using IQ-TREE software (Kalyaanamoorthy et al. 2017; Hoang et al. 2018). The results indicated that *C. intermedia*, *Cryptogramma acrostichoides* and *Llavea cordifolia* formed a strongly supported clade and constituted Cryptogrammoideae. This was in consistent with previous molecular studies (Zhang et al. 2003; Robison et al. 2018).

**Figure 1.**
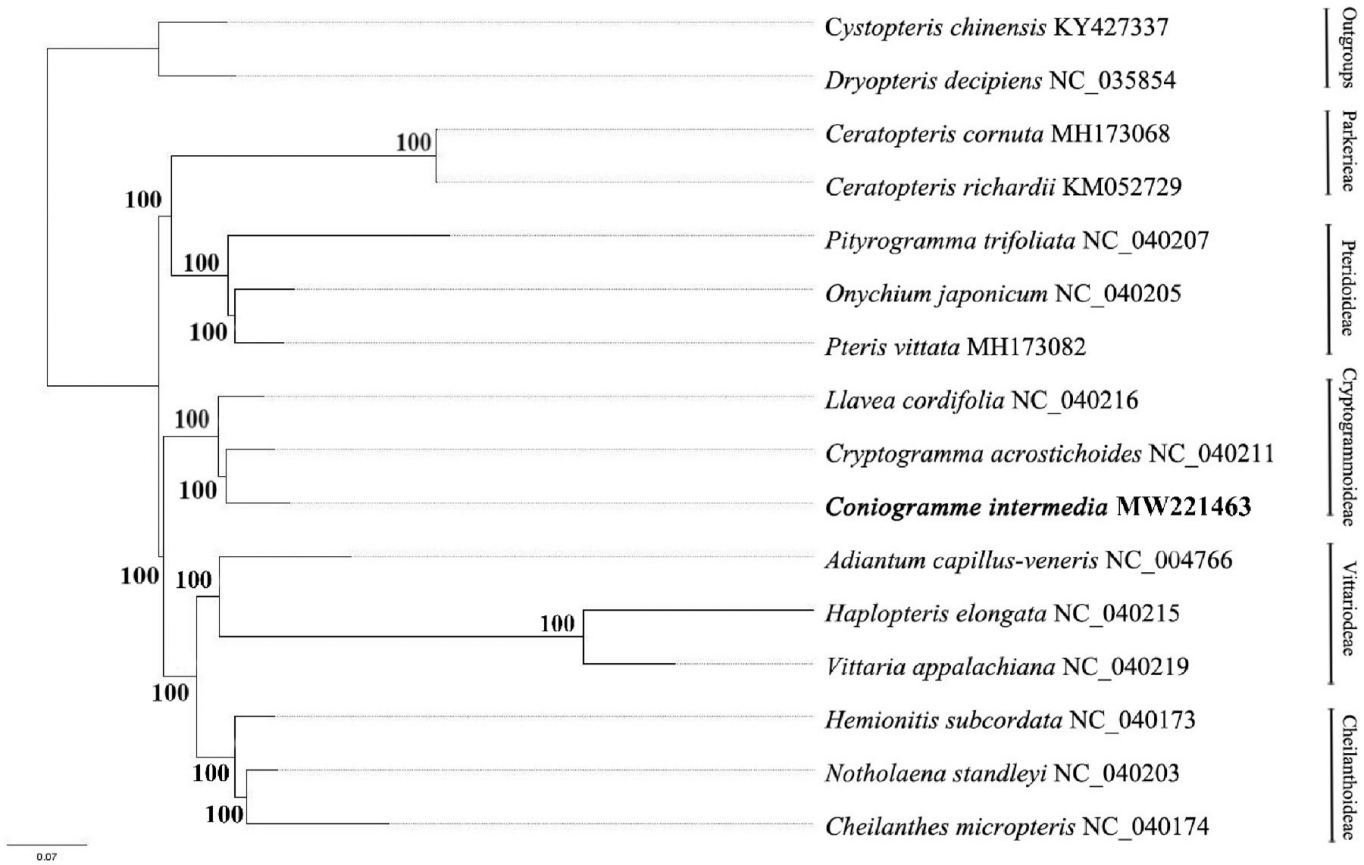
Phylogram inferred from the complete chloroplast genome sequences.

## Data Availability

The data that support the findings of this study are openly available in GenBank of NCBI at https://www.ncbi.nlm.nih.gov, reference number MW221463. Raw Illumina data is available at the Sequence Read Archive (SRA) under accession SRR12998627.

## References

[CIT0001] Christenhusz MJM, Zhang XC, Schneider H. 2011. A linear sequence of extant families and genera of lycophytes and ferns. Phytotaxa. 19(1):7–54.

[CIT0002] Doyle JJ, Doyle JL. 1987. A rapid DNA isolation procedure for small quantities of fresh leaf tissue. Phytochem Bull. 19:11–15.

[CIT0003] Fraser-Jenkins CR. 2008. Taxonomic revision of three hundred Indian subcontinental pteridophytes with a revised census-list, a new picture of fern-taxonomy and nomenclature in the Indian subcontinent. Dehra Dun (India): Bishen Singh Mahendra Pal Singh; p. 685.

[CIT0004] Hoang DT, Chernomor O, von Haeseler A, Minh BQ, Vinh LS. 2018. UFBoot2: improving the ultrafast bootstrap approximation. Mol Biol Evol. 35(2):518–522. 29077904 10.1093/molbev/msx281 PMC5850222

[CIT0005] Kalyaanamoorthy S, Minh BQ, Wong TKF, von Haeseler A, Jermiin LS. 2017. ModelFinder: fast model selection for accurate phylogenetic estimates. Nat Methods. 14(6):587–589. 28481363 10.1038/nmeth.4285 PMC5453245

[CIT0006] Katoh K, Rozewicki J, Yamada KD. 2017. MAFFT online service: multiple sequence alignment, interactive sequence choice and visualization. Bioinformatics. 20(4):1160–1166. 10.1093/bib/bbx108 PMC6781576 28968734

[CIT0007] Kearse M, Moir R, Wilson A, Stones-Havas S, Cheung M, Sturrock S, Buxton S, Cooper A, Markowitz S, Duran C, et al. 2012. Geneious Basic: an integrated and extendable desktop software platform for the organization and analysis of sequence data. Bioinformatics. 28(12):1647–1649. 22543367 10.1093/bioinformatics/bts199 PMC3371832

[CIT0008] Liu HJ, He J, Ding CH, Lyu RD, Pei LY, Cheng J, Xie L. 2018. Comparative analysis of complete chloroplast genomes of *Anemoclema*, *Anemone*, *Pulsatilla*, and *Hepatica* revealing structural variations among genera in tribe Anemoneae (Ranunculaceae). Front Pl Sci. 9:1097. 10.3389/fpls.2018.01097 PMC6073577 30100915

[CIT0009] Lu SG. 2001. A taxonomic revision of *Coniogramme* Fée (Hemionitidaceae) in Yunnan. Guihaia. 21:37–42.

[CIT0010] PPG I. 2016. A community-derived classification for extant lycophytes and ferns. J Syst E. 54:563–603.

[CIT0011] Qu XJ, Moore MJ, Li DZ, Yi TS. 2019. PGA: a software package for rapid, accurate, and flexible batch annotation of plastomes. Plant Methods. 15(1):50. 31139240 10.1186/s13007-019-0435-7 PMC6528300

[CIT0012] Robison TA, Grusz AL, Wolf PG, Mower JP, Fauskee BD, Sosa K, Schuettpelz E. 2018. Mobile elements shape plastome evolution in ferns. Genome Biol Evol. 10(10):2558–2571. 30165616 10.1093/gbe/evy189 PMC6166771

[CIT0013] Zhang GM, Ranker TA. 2013. *Coniogramme* Fée. In: Wu ZY, Raven PH, Hong DY, editors. Flora of China. Vol. 2–3. Beijing (China): Science Press; p. 171–178.

[CIT0014] Zhang GM, Zhang XC, Chen ZD. 2003. Phylogeny of cryptogrammoid ferns and related taxa based on *rbc*L sequences. Nord J Bot. 23(4):485–493.

